# Elastomeric, bioadhesive and pH-responsive amphiphilic copolymers based on direct crosslinking of poly(glycerol sebacate)-*co*-polyethylene glycol†

**DOI:** 10.1039/d2bm01335e

**Published:** 2022-11-01

**Authors:** Mina Aleemardani, Michael Zivojin Trikić, Nicola Helen Green, Frederik Claeyssens

**Affiliations:** Biomaterials and Tissue Engineering Group, Department of Materials Science and Engineering, Kroto Research Institute, The University of Sheffield Sheffield S3 7HQ UK f.claeyssens@sheffield.ac.uk; Insigneo Institute for in Silico Medicine The Pam Liversidge Building Sir Robert Hadfield Building Mappin Street Sheffield S1 3JD UK

## Abstract

Poly(glycerol sebacate) (PGS), a synthetic biorubber, is characterised by its biocompatibility, high elasticity and tunable mechanical properties; however, its inherent hydrophobicity and insolubility in water make it unsuitable for use in advanced biomaterials like hydrogels fabrication. Here, we developed new hydrophilic PGS-based copolymers that enable hydrogel formation through use of two different types of polyethylene glycol (PEG), polyethylene glycol (PEG2) or glycerol ethoxylate (PEG3), combined at different ratios. A two-step polycondensation reaction was used to produce poly(glycerol sebacate)-*co*-polyethylene glycol (PGS-*co*-PEG) copolymers that were then crosslinked thermally without the use of initiators or crosslinkers, resulting in PGS-*co*-PEG2 and PGS-*co*-PEG3 amphiphilic polymers. It has been illustrated that the properties of PGS-*co*-PEG copolymers can be controlled by altering the type and amount of PEG. PGS-*co*-PEG copolymers containing PEG ≥ 40% showed high swelling, flexibility, stretching, bioadhesion and biocompatibility, and good enzymatic degradation and mechanical properties. Also, the addition of PEG created hydrogels that demonstrated pH-responsive behaviours, which can be used for bioapplications requiring responding to physicochemical dynamics. Interestingly, PGS-*co*-40PEG2 and PGS-*co*-60PEG3 had the highest shear strengths, 340.4 ± 49.7 kPa and 336.0 ± 35.1 kPa, and these are within the range of commercially available sealants or bioglues. Due to the versatile multifunctionalities of these new copolymer hydrogels, they can have great potential in soft tissue engineering and biomedicine.

## Abstract 2

Novel PGS-*co*-PEG elastomers showed multifunctional characteristics such as high swelling, flexibility, bioadhesiveness and biocompatibility, and good biodegradation, mechanical properties and pH-responsive behaviour.

## Graphical abstract

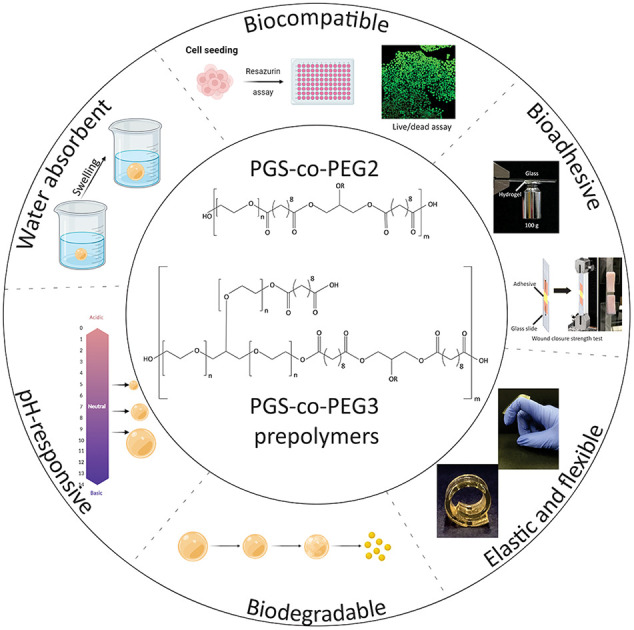

## 1. Introduction

Poly(glycerol sebacate) (PGS) is a tough, biocompatible, enzymatically degradable, and thermoset elastomer,^1^ which was introduced as a novel biopolymer for the first time by the Langer Group for tissue engineering applications in 2002.^2^ A polycondensation reaction between glycerol and sebacic acid yields a meltable and soluble pre-polymer (pre-PGS), which is subsequently crosslinked to produce a polymer network (PGS).^3,4^ One of the most attractive features of PGS is that it can be easily modified by adjusting the synthesis parameters, such as the ratio of glycerol to sebacic acid, synthesis time and temperature, which allows for precise control over the mechanical and degradation properties. Other advantages of PGS are the biologically relevant range of its mechanical properties; the Young's modulus of PGS is between 0.025 and 1.2 MPa, which can be used for application in different soft tissues in particular.^5,6^ The raw materials, glycerol and sebacic acid, are cheap and degradable in physiological conditions, leading to Food and Drug Administration (FDA) approval as a biomedical material.^7^ Additionally, surface-erodible PGS has linear weight loss with a linear degradation process that makes it suitable for long-term implantation^8^ as well as the controlled release of bio/molecules when loaded into the polymer matrix.^9,10^ Even though PGS is highly desirable, there are still challenges associated with it, such as its limited hydrophilicity and high curing temperatures.^11,12^ In the structure of PGS, there are hydroxyl groups that allow the incorporation of various functionalities, thus permitting its physiochemical characteristics to be tailored. Therefore, it is possible to modify PGS physically and chemically to improve its properties. Hydration properties are vital to achieving optimum features like biocompatibility, biodegradability, and mechanical behaviour, and hydrophilic moieties can provide these attributes.^3,12^ Adding a polyethylene glycol (PEG) segment to the PGS backbone to produce a PGS-*co*-PEG copolymer decreases hydrophobicity, making it more suitable for biomedical applications. It has been demonstrated that by altering the amount of PEG in the copolymer, characteristics such as Young's modulus, degradation rate, and water uptake can be regulated.^13,14^ Another advantage of PGS-*co*-PEG is that with some modifications, it can be crosslinked to develop hydrogels under ambient conditions.^15^ In addition, the PEG in the structure allows it to be modified and used in different ways.^12,16^ PEG is an FDA approved polymer with hydrophilic, non-toxic, and non-immunogenic properties. There are different types of PEGs composed of polyether compounds repeating ethylene glycol units based on the constituent monomer or parent molecules, such as ethylene glycol, ethylene oxide, or oxyethylene. These oligomers and polymers are usually referred to as PEGs when their molecular masses are ≤20 000 g mol^−1^.^17,18^ To date, only PEG 2-arm, with two functional groups, has been used to create a PGS-*co*-PEG copolymer. Therefore, the main aim of this study is to evaluate PGS-*co*-PEG copolymers formed using two kinds of PEG with different reactant ratios.

Hydrogels are of interest in many different fields, including biomedicine and biotechnology, due to their macromolecular network structure and high water content, which also makes them suitable for loading biomolecules.^19^ There are various strategies to develop hydrogels, but generally, they have a three-dimensional (3D) polymer mesh structure or network that absorbs considerable amounts of water and retains their swollen state by stabilisation *via* non-covalent bonding of water within their interstices.^20,21^ Hydrogels can be made with natural and synthetic polymers and used for different biomedical applications such as 3D cell culture, tissue engineering, and drug delivery.^22^ Usually, synthetic polymers provide better reproducibility and higher mechanical strength but lower biocompatibility than natural polymers. Therefore, there is a great interest in improving the biocompatibility of synthetic polymers while retaining their beneficial aspects for biomedical applications.^23^ To mimic the physiochemical dynamics of the human body, polymers and hydrogels must replicate native tissue as closely as possible. Hydrogels with elastomeric mechanical features, for instance, are excellent choices for soft tissue engineering, with biomimetic and synchronous deformations that are similar to native tissue dynamics.^24,25^ There have also been many studies using the changes associated with *in vivo* implantation of polymers and hydrogels, such as changes in temperature, pH, ionic strength and mechanical stress, to increase the diffusion properties of for controlled drug delivery,^26,27^ and these responsive materials are increasingly used as smart materials for soft tissue engineering.^28–30^

This study systematically analyses the effect of PEG type and ratios on the physicochemical and biological features of the resulting copolymers. Herein, we demonstrate novel bioadhesive, elastomeric, pH-responsive, enzymatically degradable and biocompatible hydrogel systems based on the copolymers of PGS, PEG 2-arm (polyethylene glycol 1000 or PEG2) and PEG 3-arm (glycerol ethoxylate 1000 or PEG3).

## 2. Materials and methods

### 2.1. Materials

Sebacic acid (SA), polyethylene glycol 1000 termed PEG2 (*M*_w_ = 1000 g mol^−1^), glycerol ethoxylate termed PEG3 (*M*_w_ = 1000 g mol^−1^), glycerol, tetrahydrofuran (THF), Dulbecco's modified Eagle's medium (DMEM), amphotericin B, fetal bovine serum (FBS), penicillin/streptomycin (PS), l-glutamine, trypsin, paraformaldehyde, lipase, dimethyl sulfoxide (DMSO) and ethanol were all purchased from Sigma Aldrich. CellTrace™ Calcein Green, AM (20 × 50 μg) and ethidium bromide (10 mg mL^−1^) were purchased from ThermoFisher Scientific.

### 2.2. Preparation of poly(glycerol sebacate)-*co*-poly(ethylene glycol) (PGS-*co*-PEG) copolymers

**Fig. 1:**
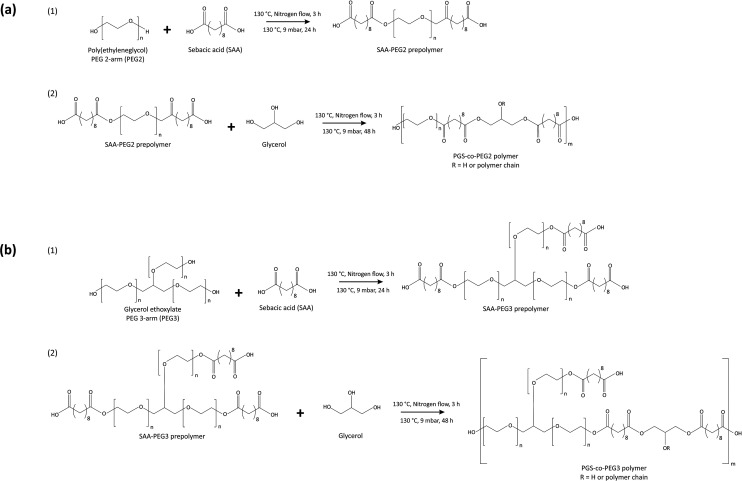
Synthesis scheme for producing (a) PGS-*co*-PEG2 and (b) PGS-*co*-PEG3 copolymers.

The copolymerisation of the PGS-*co*-PEG pre-polymer was conducted in the following two steps: (1) polycondensation of sebacic acid (SA) and PEG with different types and weight ratios to yield the SA/PEG pre-polymer, and (2) addition of glycerol and synthesis of PGS-*co*-PEG pre-polymers. In order to synthesise the PGS-*co*-PEG prepolymers in this study, two types of PEG were used, PEG 2-arm or PEG2 and PEG 3-arm or PEG3 (Table 1). First, the polycondensation of SA and PEGs, included at 20%, 40% or 60% wt was performed under stirring conditions, at 130 °C, under nitrogen flow for 3 h and then a vacuum of 9 mbar for a further 24 h. In the case of PEG2, firstly, it was melted in a vacuum chamber at 90 °C. Second, glycerol was added and mixed completely under the nitrogen flow, and the reaction was continued at 130 °C under a vacuum of 9 mbar for 48 h (Fig. 1). The resulting pre-polymer resins were viscous. To prepare fully cured copolymer films, pre-polymer solutions were evenly distributed onto polytetrafluoroethylene substrates (Thermo Fisher Scientific, UK) and thermally cured under vacuum at 130 °C for 72 h.

**Table 1:** Ratios of the PGS-*co*-PEG (either PEG2 or PEG3) and their methacrylation pre-polymers

Sample code		Molar ratio (PEG : sebacic acid : glycerol)	PEG (wt%)
PEG 2-arm	PEG 3-arm		
PGS-*co*-20PEG2	PGS-*co*-20PEG3	0.07 : 1 : 1	20
PGS-*co*-40PEG2	PGS-*co*-40PEG3	0.19 : 1 : 1	40
PGS-*co*-60PEG2	PGS-*co*-60PEG3	0.44 : 1 : 1	60

### 2.3. Characterisation of PGS-*co*-PEG prepolymers using gel permeation chromatography (GPC)

The molecular weights of the prepolymer of PGS and PGS-*co*-PEG copolymers were determined using gel permeation chromatography (GPC) (Viscotek GPC Max VE 2001) with a differential refractive index detector (Waters 410). The specimens were dissolved in THF (0.10 mg mL^−1^) and injected at a flow rate of 1 mL min^−1^. Polystyrene standards were used as a calibrator, and toluene was added as a reference (0.2% of toluene was added to the samples/standards). Samples were analysed at 40 °C by a 650 mm PLgel 5 μm mixed C column. Chromatogram peaks were analysed to deduce the prepolymer molecular weights as number average (*M*_n_), weight average (*M*_w_) and the polydispersity index (PDI).

### 2.4. Characterisation of PGS-*co*-PEG prepolymers by proton nuclear magnetic resonance (NMR) spectroscopy

PGS and PGS-*co*-PEG prepolymers were analysed through proton (^1^H) nuclear magnetic resonance (NMR) spectroscopy (Burker AVIIIHD 400 NMR spectrometer) at 400 MHz, standard ^1^H experiments were recorded using a 30° pulse for excitation, 64k acquisition points over a spectral width of 20.5 ppm with 64 transients and a relaxation delay of 2 s. Prepolymer specimens were dissolved in 1 mL of deuterated chloroform (CDCl_3_) at 1% (w/v). Chemical shifts were referenced to CDCl_3_ at 7.27 ppm. Spectra were analysed using MestReNova software (Mestrelab Research).

### 2.5. Characterisation of PGS-*co*-PEG prepolymers using attenuated total reflectance-Fourier transform infrared (ATR-FTIR) spectroscopy

Attenuated total reflectance-Fourier transform infrared (ATR-FTIR) spectroscopy was done using a PerkinElmer Spectrum One NTS analyser with readings between 600–4000 cm^−1^, 4 cm^−1^ resolution, 16 scans.

### 2.6. Solubility and sol–gel content evaluation

The solubility of PGS-*co*-PEG pre-polymers and cured copolymer samples (∼1 g) was evaluated by immersing the specimens in various solvents (5 mL), namely, tetrahydrofuran (THF), dichloromethane (DCM), methanol, ethanol, acetone and phosphate-buffered saline (PBS) for 48 h with stirring at 37 °C.

To determine the degree of crosslinked network of cured samples, sol–gel content analysis was conducted. PGS-*co*-PEG hydrogels (7 mm in diameter, 1.2–1.6 mm thickness and *n* = 5) were weighed (*W*_0_) and then were allowed to swell in tetrahydrofuran (THF) and phosphate-buffered saline (PBS) for 48 h. After complete drying, specimens were weighed (*W*_d_), and the percentage of sol contents was calculated by eqn (1).

1 Sol (%) = [(*W*_0_ − *W*_d_)/*W*_0_] × 100

### 2.7. Water contact angle evaluation

The water contact angle of the PGS-*co*-PEG specimens was measured by Goniometer FTÅ 200 and First Ten Angstroms (FTA) Software (UK). A water droplet with the needle was a 30 gauge (blunt-end syringe needle) was dosed onto the sample surface. Angle measurements were carried out using a digital camera and FTA software after 10 seconds.

### 2.8. pH-Responsive swelling and enzymatic degradation evaluation

The specimens (diameter of 7 mm, thickness of 1.2–1.5 mm and *n* = 5) were first weighed (*W*_dry_) and, secondly, fully immersed in PBS at pH = 5 (citrate buffer), 7.4 and 9.1 (glycine–NaOH buffer), and incubated at physiological temperature, 37 °C. The swollen samples were collected at specific time intervals, filter paper used to remove excess surface PBS, and then weighed (*W*_wet_). The swelling ratio was determined by eqn (2).

2 Swelling ratio (%) = [(*W*_wet_ − *W*_dry_)/*W*_dry_] × 100

The degradation behaviour of the PGS-*co*-PEG hydrogels was examined *in vitro*. The samples (diameter of 7 mm, thickness of 1.2–1.4 mm and *n* = 5) were weighed (*W*_ini_), then incubated at 37 °C in the following degradation media: (1) PBS and (2) PBS with lipase (110 U L^−1^). The media was changed daily to maintain enzyme activity. To determine the weight loss, at specific time intervals, over 35 days, the specimens were washed and dried before being weighed (*W*_day_). The weight loss (%) was measured by eqn (3).

3 Weight loss (%) = [(*W*_ini_ − *W*_day_)/*W*_ini_] × 100

### 2.9. Poly(ethylene glycol) (PEG2) and glycerol ethoxylate (PEG3) release study

For the quantitative analysis of PEG2 and PEG3 release, a two-phase system colourimetric assay was used.^31,32^ Standard curves were first obtained through serial dilution of a stock solution (20 mg mL^−1^ of either PEG2 or PEG3 in PBS). To prepare the ammonium ferrothiocyanate reagent, 16.2 g of anhydrous ferric chloride (FeCl_3_) and 30.4 g of ammonium thiocyanate (NH_4_SCN) were dissolved in 1 L of distilled water. In a microfuge tube, aliquots of 0.5 mL each of ammonium ferrothiocyanate reagent (upper phase) and chloroform (bottom phase) were taken. The PGS-*co*-20PEG2, PGS-*co*-40PEG2, PGS-*co*-20PEG3, PGS-*co*-40PEG3 and PGS-*co*-60PEG3 hydrogels (7 mm of diameter and average of 1.4–1.9 mm thickness) were soaked in PBS at 37 °C. At specific intervals over 35 days, aliquots (50 μl) of the samples were added to the previously prepared two-phase system (in microfuge tubes). After vigorous mixing on a vortex and rocker for 30 min, the tubes were centrifuged at 5590*g* for 2 min (Sanyo MSE Micro Centaur MSB010.CX2.5, UK). Afterwards, the lower chloroform layers were removed, and their absorbance was read at 510 nm using a UV Spectrophotometer (JENWAY 6305). The release medium was collected at each time and replenished with fresh PBS, and PGS was used as a control. The replication of each sample was 5 (diameter of 7 mm, thickness of 1.2–1.4 mm and *n* = 5).

### 2.10. Uniaxial tensile test

The mechanical properties of PGS-*co*-PEG specimens were determined using a MultiTest-dV tester (Mecmesin, Slinfold, UK), using a modified version of ASTM D638-10. PGS-*co*-PEG copolymers, PGS-*co*-PEG2 and PGS-*co*-PEG3, were thermally cured at 130 °C for 3 days in a PTFE petri dish and laser-cut (HPC LASER LS 3040, LASERSCRIPT) into dog-bone-shaped tensile test specimens following ASTM D638-10. Samples were tested on a MultiTest-dV tester using a load cell of 25 N, grip distance of 10 mm and extension rate of 1 mm s^−1^, and both force and elongation data were recorded. The Young's modulus was calculated by the linear-elastic region of the stress–strain curve of each sample. The maximum elongation or elongation at yield is defined as the percentage elongation at the end of the linear-elastic area. The thickness of the samples was measured as 1–1.3 mm, and each sample type was analysed 5 times (*n* = 5).

### 2.11. Lap-shear strength

The shear strength of PGS-*co*-PEG samples was measured to examine bioadhesive properties through a modified lap-shear test based on the F2255-05 ASTM standard.^33^ Two glass slides (10 mm × 50 mm, coated with a 20% w/v gelatin solution at 37 °C and dried at room temperature) were used, and specimens with 10 mm × 10 mm dimension and 1–1.3 mm thickness were placed between them. Tensile stress was applied (1 mm min^−1^) using a MultiTest-dV tester (Mecmesin, Slinfold, UK), and the shear strengths of the samples were measured at the detachment point (*n* ≥ 5). Dermabond™ Mini Topical Skin Adhesive was used as a control.

### 2.12. Wound closure strength test

The wound closure capability of PGS-*co*-PEG specimens was evaluated by a modified ASTM standard test, F2458-05.^33,34^ For this test, porcine skin was purchased from a local butcher and then cut into 10 mm × 20 mm pieces. The excess fat was removed and the skin kept hydrated in PBS before testing. A cut (all the way through) was made in the porcine skin with a straight-edge scalpel to simulate a wound, and the skin was fixed between two glass slides (20 mm × 60 mm) using superglue. The space between slides and sample (10 mm × 10 mm dimension and 1–1.3 mm thickness) was adjusted to 10 mm. Tensile loading was conducted at a 1 mm min^−1^ strain rate using a MultiTest-dV tester (*n* ≥ 5), and the adhesive strengths were recorded based on the detachment point. Dermabond™ Mini Topical Skin Adhesive was used as a control.

### 2.13. Adhesion and flexibility test

To demonstrate the adhesion of the PGS-*co*-40PEG2 and PGS-*co*-60PEG3 copolymers with different substrates and a 100 g weight scale, a circle-shaped sample with a diameter of 12 mm was bored and bonded to glass, polycaprolactone (PC), polytetrafluoroethylene (PTFE), silicone, wood and aluminium 2 × 5 cm^2^ slides, and next, a 100 g stainless steel weight was stuck to the samples. The joint was slightly pressured with a finger for 10 s.^35^

Also, in order to illustrate the flexibility of PGS-*co*-40PEG2 and PGS-*co*-60PEG3 copolymers, as well as their adhesiveness, different images in various situations have been captured.

### 2.14. Biocompatibility testing

#### 2.14.1. Cell seeding

PGS-*co*-PEG hydrogels (diameter of 9 mm) were washed in methanol and PBS then air-dried PGS-*co*-PEG samples were sterilised by immersing them in 70% ethanol for an hour, followed by a series of washes in sterile PBS (10 min, repeated three times). The specimens were then air-dried in a sterile environment for 24 h prior to soaking overnight in foetal bovine serum (FBS). Human keratinocyte (HaCat ATCC® HB-241TM) cells were maintained in adherent culture in cell culture medium (DMEM containing 10% (v/v) FBS, 100 IU mL^−1^ penicillin, 100 mg mL^−1^ streptomycin, 2 mM l-glutamine and 0.625 μg mL^−1^ amphotericin B) at 37 °C and 5% CO_2_. Cells were passaged by once flasks reached 80% confluency. HaCat cells were seeded in cell culture medium on samples at a density of 2 × 10^4^ (15 μL) per sample, which were then incubated (37 °C and 5% CO_2_) for 30 min before addition of 900 μL of the cell culture medium. Tissue culture plastic (TCP) and PGS were the controls. The cells were cultured for 1, 3 and 7 days, with the medium changing every 2 days. The replications for each sample was five (*n* = 5).

#### 2.14.2. Cell metabolic activity

Cellular metabolic activity was measured using the resazurin reduction assay, and cell viability was estimated. Resazurin solution (nonfluorescent, blue) is reduced by the cells to resorufin (fluorescent, pink), which can be detected by a fluorescent plate reader. Briefly, the resazurin working solution was made from a 1 mM resazurin stock solution diluted to 100 μM in culture medium. The scaffolds were transferred into a fresh well plate, and 1 mL of resazurin solution was added to each well and then incubated for 4 h at 37 °C. A spectrofluorometer (FLX800, BIO-TEK Instruments, Inc.) measuring excitation and emission wavelengths of 540 nm and 630 nm respectively was used to measure duplicate samples of 200 μL of the reduced solution from each scaffold. Fresh scaffold/cell constructs were used for resazurin reduction assays for each time interval.

### 2.15. Live/dead assay

#### 2.15.1. Samples preparation

PGS-*co*-PEG samples were cut into 300 μm thick discs *via* vibrotome (5100mz, Campden Instruments, Loughborough, UK). The vibrotome frequency, amplitude and speed were set at 80 Hz, 1.5 mm and 0.10 mm s^−1^, respectively. The cutting was conducted in wet conditions (deionized water was added to the water bath).

#### 2.15.2. Live/dead staining

HaCat cells were seeded in a cell culture medium for each sample at a density of 2 × 10^4^ (20 μL). Live/dead assay was conducted after three days of cell culture. Calcein green AM and ethidium bromide were prepared by diluting the stains in PBS (2 μL mL^−1^ in DMSO and 0.1 μL mL^−1^ in PBS, respectively) and incubating for 30 min in darkness. The cells were then observed under an upright confocal microscope (Zeiss LSM510-META, UK) equipped with a ×10 objective (W-N-Achroplan 10× NA 0.3, Zeiss Ltd, UK) for live and dead cells, where living cells were indicated by green colour (excitation/emission at 494/517 nm) and dead cells by red colour (excitation/emission 517/617 nm). *Z*-Stack images (512 × 512 pixels) were taken for each sample and projected to a single image.

### 2.16. Statistical analysis

The results were analysed by Origin Pro 2020 and one-way analysis of variance (ANOVA), *post hoc* and Tukey analysis and plotted as mean ± standard deviation (SD). In the figures, the *p*-values represent the statistical differences between groups. The number of replicates (*n*) is given in the Materials and methods section and figure legends.

## 3. Results and discussion

Among all the PGS-*co*-PEG groups, PGS-*co*-60PEG2 could not be thermally cured under the specified reaction condition; therefore, the results related to this sample cannot be reported in some cases. This phenomenon could be due to a failure to crosslink in these conditions because the percentage of PEG is high, and the number of free –OH groups available for crosslinking is low. Similarly, this case was reported in another study where, within the copolymer structure (40PEGS-0.67C/H group), there was a lack of free –OH and high PEG amount.^36^

### 3.1. Gel permeation chromatography (GPC) analysis

**Fig. 2:**
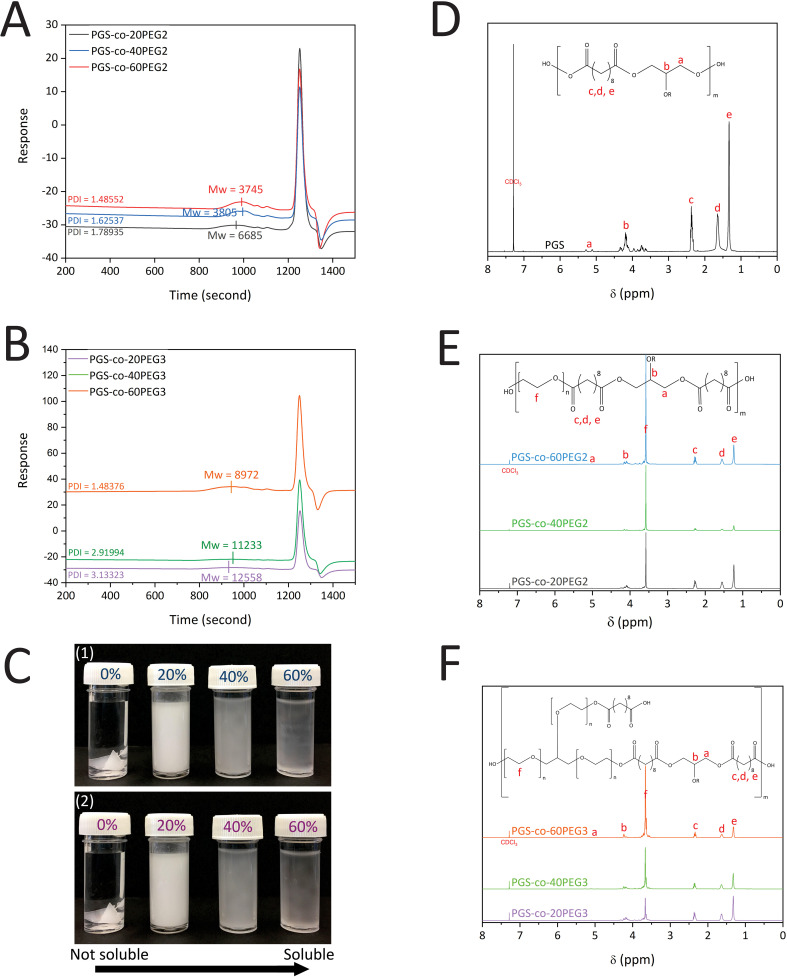
(A) GPC curves of PGS-*co*-PEG2, (B) GPC curves of PGS-*co*-PEG3 with different concentrations of PEG, 20%, 40% and 60% wt, (C) Images of (1) PGS-*co*-PEG2 and (2) PGS-*co*-PEG3 illustrating water solubility at various concentrations of PEG, 0%, 20%, 40% and 60% wt, ^1^H-NMR spectra of (D) PGS polymer, (E) PGS-*co*-PEG2 copolymers and (F) PGS-*co*-PEG3 copolymers with different concentrations of PEG, 20%, 40% and 60% wt.

PGS-*co*-PEG prepolymers were synthesised *via* the following two polycondensation steps: (1) reaction of SA with PEG at 130 °C for 24 h and (2) reaction of SA-PEG prepolymer with glycerol at 130 °C for 48 h. The concentration (20%, 40% and 60% wt) and type of the PEG (PEG2 and PEG3) were varied; GPC was performed to determine the effect on the *M*_n_, *M*_w_ and PDI of the resulting PGS-*co*-PEG prepolymer. The results indicate that the *M*_w_ of PGS was 8275 g mol^−1^ (DI = 2.83). The addition of 20%, 40% and 60% PEG2 resulted in copolymers with *M*_w_ of 6685 g mol^−1^ (PDI = 1.79), 3805 g mol^−1^ (PDI = 1.63) and 3745 g mol^−1^ (PDI = 1.49), respectively, showing close control over the condensation reaction (Fig. 2A). For samples with PEG3, the addition of 20%, 40%, and 60% PEG made copolymers with *M*_w_ of 12 558 g mol^−1^ (PDI = 3.13), 11 233 g mol^−1^ (PDI = 2.91) and 8972 g mol^−1^ (PDI = 1.48), respectively (Fig. 2B). The addition of either PEG2 or PEG3 resulted in a decline in PGS-*co*-PEG molecular weight; however, due to the structure of PEG3 (glycerol ethoxylate), PGS-*co*-PEG3 specimens have higher molecular weights than PGS-*co*-PEG2 copolymers. By adding more PEG, the degree of esterification decreases, which results in prepolymers with lower molecular weights.^36^

### 3.2. Proton nuclear magnetic resonance (NMR) analysis

The successful synthesis of PGS, PGS-*co*-PEG2 and PGS-*co*-PEG3 copolymers was confirmed by ^1^H NMR analysis (Fig. 2D–F). Signals around 1.30 ppm (e), 1.5–1.6 ppm (d) and 2.2–2.3 ppm (c) were identified as the methylene protons (–COCH̲_2_CH̲_2_CH̲_2_–) of SA. Peaks between roughly 4.1–4.2 and 5.2–5.3 ppm (b and a) were identified as the methylene protons (–CH̲_2_CH̲–) of glycerol. The incorporation of the PEG segment was confirmed by the presence of the methylene peak appearing (f) in the spectra of PGS-*co*-20PEG2 (at 3.70 ppm), PGS-*co*-40PEG2 (at 3.65 ppm), PGS-*co*-60PEG2 (at 3.61 ppm), PGS-*co*-20PEG3 (at 3.71 ppm), PGS-*co*-40PEG3 (at 3.70 ppm) and PGS-*co*-60PEG3 (at 3.67 ppm).^15,36,37^ Based on the integration of methylene peaks of PEG and SA,^37,38^ the actual PEG percentage within PGS-*co*-20PEG2, PGS-*co*-40PEG2 and PGS-*co*-60PEG2 and PGS-*co*-20PEG3, PGS-*co*-40PEG3 and PGS-*co*-60PEG3 was determined (Table 2). The correlation between the theoretical PEG ratio and the ratio calculated from ^1^H NMR (experimental ratio) was closely aligned with no significant differences, which demonstrates that the copolymer synthesis was controlled precisely.

**Table 2:** The theoretical and experimental ratio of PEG percentages within PGS-*co*-PEG2 and PGS-*co*-PEG3 copolymers

Polymer	Theoretical PEG%	Calculated PEG%
PGS	0	0
PGS-*co*-20PEG2	20	19
PGS-*co*-40PEG2	40	39
PGS-*co*-60PEG2	60	61
PGS-*co*-20PEG3	20	22
PGS-*co*-40PEG3	40	41
PGS-*co*-60PEG3	60	62

### 3.3. Attenuated total reflectance-Fourier transform infrared (ATR-FTIR) analysis

**Fig. 3:**
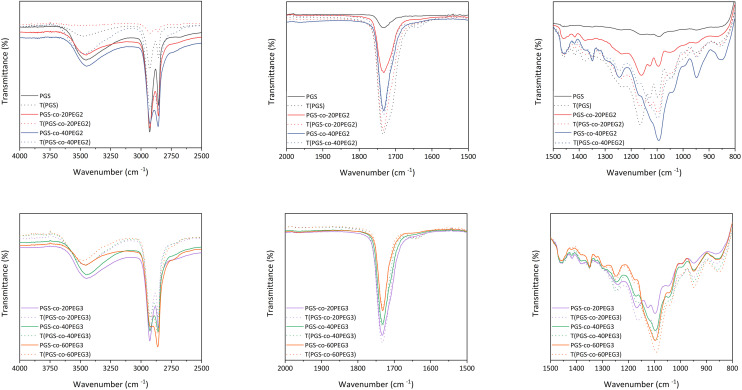
The ATR-FTIR spectra of PGS, PGS-*co*-PEG2 and PGS-*co*-PEG3 polymers were obtained before and after the thermal crosslinking process.

ATR-FTIR confirmed the synthesis of the PGS pre-polymer (Fig. 3) with the strong peaks at 1173 cm^−1^ and 1733 cm^−1^, presenting the ester linkage formations between hydroxyl groups (–OH) from glycerol and carboxylic groups (–COOH) from sebacic acid (SA). In order to fully crosslink PGS prepolymer and PGS-*co*-PEG copolymers, thermal curing was used by subjecting the prepolymer solutions to 130 °C for 72 h.^1^ ATR-FTIR was used to evaluate the impact of thermal crosslinking on the copolymer chains before and after curing (Fig. 3). The free hydroxyl groups (–OH) available on the PGS backbone react with unreacted sebacic acid (SA) to form crosslinked networks; also, for PGS-*co*-PEG3 copolymers, there is another free –OH present on PEG 3-arm segment, which can react and result in further crosslinking (Fig. 1). As Fig. 3 illustrates, in PGS prepolymer, PGS-*co*-PEG2 and PGS-*co*-PEG3 copolymers, the hydroxyl peaks between 3300 to 3700 cm^−1^ decreased after the thermal crosslinking process. Further, by adding PEG2, as well as PEG3, this reduction was reinforced. Asymmetric and symmetric stretching vibrations of C–H bonds were detected at almost 2927 and 2861 cm^−1^, respectively, and after thermal crosslinking, the intensity of the peaks was reduced. As a result of thermal curing, the ester bond peaks increased in intensity, showing the formation of new ester bonds between polymer chains. Ester bonds within the prepolymers were demonstrated by firm peaks at ∼1733 cm^−1^ (C=O), 1159 cm^−1^ and 1095 cm^−1^ (C–O). When compared with the PGS prepolymer spectrum, a band around 1120 cm^−1^ (C–O–C) was observed in the PGS-*co*-PEG copolymers that related to symmetric stretching vibrations.^38^ Moreover, other peaks near 1150–1160 cm^−1^ were associated with the ether bonds in both PEG2 and PEG3, which were segmented successfully into the PGS-*co*-PEG backbone. In PGS-*co*-PEG2 and PGS-*co*-PEG3, there was an absorption peak of C–O–C symmetric stretching vibration at 950 cm^−1^, suggesting PGS-*co*-PEG has a more hydrophilic ester bond than PGS.^39^

### 3.4. Solubility and sol–gel content

Increasing the ratio of either PEG2 or PEG3 enhanced the copolymer dissolution in water. In fact, PGS-*co*-PEG is no longer a hydrophobic polymer, instead, it is an amphiphilic polymer; the PGS confers the hydrophobic segment, while PEG is the hydrophilic part (Fig. 2C). Moreover, with no curing, the PGS-*co*-PEG pre-polymers were soluble in THF, DCM, methanol, ethanol, acetone and PBS. Further, the pre-polymers were re-meltable and deformable into the desired form; and hence, they can be used to fabricate various scaffold structures for tissue engineering applications (Fig. 7C).

The sol–gel content assay was used to investigate the crosslinking density in the polymer. Due to the addition of PEG, it was anticipated that the crosslinking density would decrease.^38,40^ The sol content, or unreacted pre-polymer, within the covalently crosslinked PGS-*co*-PEG polymer network was determined through swelling the network in both PBS and THF. Within THF, the swelling degree of copolymers is high, allowing the sol to diffuse out. At the same time, PBS is a standard component of biological assays, so we also determined the sol concentration in PBS. The sol content of PGS was 0 in PBS and 0.1 ± 1.76 in THF within 48 h, which is expected since there is no PEG in the structure. In Table 3, the sol content of PGS-*co*-PEG2 and PGS-*co*-PEG3 samples both in PBS and THF are given. The percentage sol content of PGS-*co*-PEG2 in THF was similar to the percentage of PEG within the hydrogel; for PGS-*co*-PEG3, the percentage of sol content was approximately half the percentage of PEG in the hydrogel structure. Forming a covalently crosslinked network is correlated with the presence of free hydroxyl groups (–OH groups) on the biopolymer backbone (Table 3 and Fig. 1), and there are more free –OH groups in PGS-*co*-PEG3 compared with PGS-*co*-PEG2. However, the PEG segment reduces hydroxyl content in the PGS-*co*-PEG backbone and results in reaction barriers (crosslinking process).^36,40^ This was evident through an increase in the sol contents of PGS-*co*-PEG2 and PGS-*co*-PEG3 copolymers compared to PGS.

**Table 3:** Sol content of PGS-*co*-PEG2 and PGS-*co*-PEG3 in PBS and THF (*n* = 5)

Sample code (PGS-*co*-PEG2)	Sol content (%)		Sample code (PGS-*co*-PEG3)	Sol content (%)	
	PBS	THF		PBS	THF
PGS-*co*-20PEG2	7.29 ± 0.28	19.37 ± 0.73	PGS-*co*-20PEG3	2.61 ± 0.30	9.66 ± 1.60
PGS-*co*-40PEG2	18.25 ± 0.57	35.47 ± 1.08	PGS-*co*-40PEG3	5.98 ± 0.41	19.11 ± 2.06
			PGS-*co*-60PEG3	17.09 ± 0.99	33.85 ± 1.39

### 3.5. Water contact angle

**Fig. 4:**
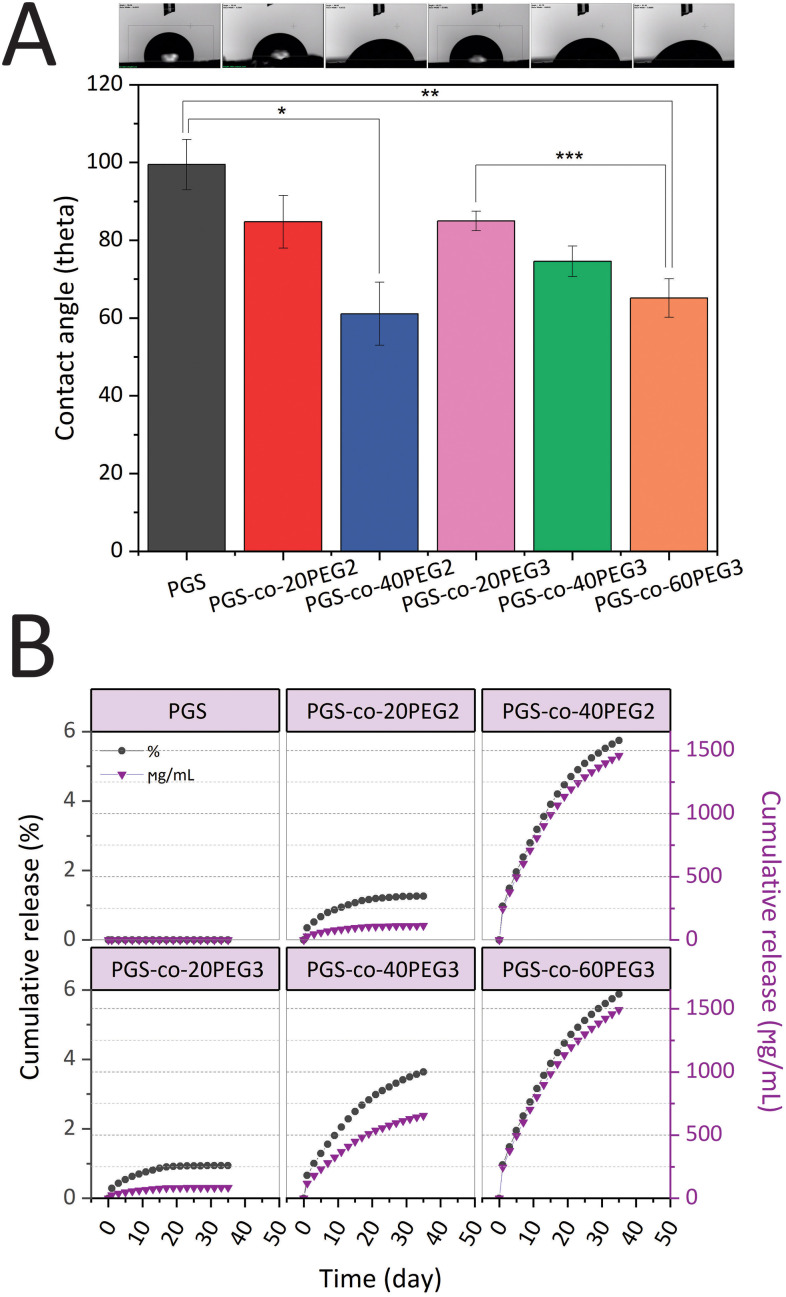
(A) The surface water contact angle of PGS-*co*-PEG copolymers. The images illustrate the water droplet shapes on the surface of PGS-*co*-PEG specimens (*,**, *** *p*-value <0.01) (*n* = 5) and (B) results of cumulative PEG release of PGS-*co*-20PEG2, PGS-*co*-40PEG2, PGS-*co*-20PEG3, PGS-*co*-40PEG3 and PGS-*co*-60PEG3 hydrogels and control (PGS) over 35 days (*n* = 5). The cumulative results of the PEG release are expressed in percentage (grey circles) and μg mL^−1^ (purple triangles).

PGS-*co*-PEG copolymers were examined for their hydration properties in both surface and bulk. By measuring the water contact angles on the surface of specimens, the surface hydration properties were investigated (Fig. 4A). It is possible to correlate contact angle with hydrophilicity or hydrophobicity, and a higher angle indicates a less wettable surface and thus a higher hydrophobicity. Adding PEG2 and PEG3 to the PGS-*co*-PEG copolymers increased the surface wettability and decreased the water contact angles. As shown in Fig. 4A, the water contact angle of pure PGS was 99.46 ± 6.50°, but after adding PEG, the angle was reduced to 61.09 ± 8.09° and 65.15 ± 4.96° for PGS-*co*-40PEG2 and PGS-*co*-60PEG3, respectively. Due to their enhanced surface wettability, PGS-*co*-PEG2 and PGS-*co*-PEG3 copolymers could be useful for tissue engineering applications since cells prefer hydrophilic surfaces to adhere to and proliferate on.^41^

### 3.6. pH responsive swelling and enzymatic degradation

#### 3.6.1. Swelling in PBS at pH = 7.4

**Fig. 5:**
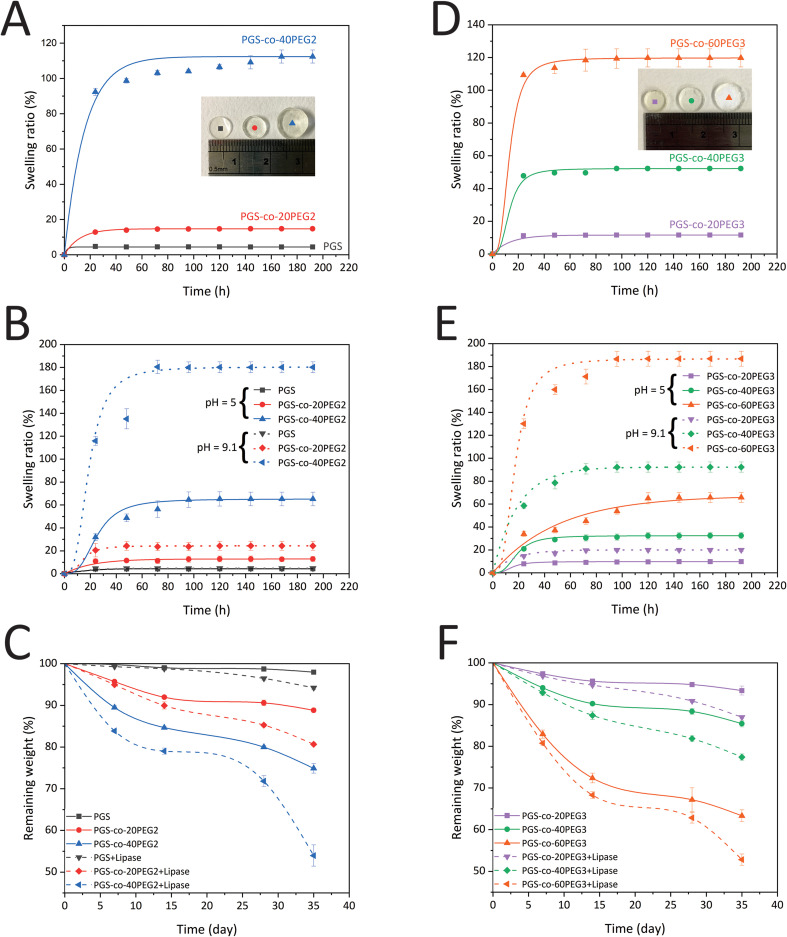
Hydrogel mass profiles of PGS-*co*-PEG. (A) The hydration kinetics of PGS and PGS-*co*-PEG2 copolymers, determined over the course of 192 h (*n* = 5), with a notably high swelling ratio for PGS-*co*-40PEG2. (B) The pH-responsive water swelling ratios of PGS-*co*-PEG2 hydrogels were measured at pH 5.0 and pH 9.1 over 192 h (*n* = 5). (C) The degradation kinetics of PGS-*co*-PEG2 copolymers within PBS with and without lipase over 35 days (*n* = 5), (D) the hydration kinetics of PGS-*co*-PEG3 copolymers measured over 192 h (*n* = 5), (E) the pH-responsive water swelling ratios of PGS-*co*-PEG3 hydrogels were measured at pH 5.0 and pH 9.1 over 192 h (*n* = 5) and (F) the degradation kinetics of PGS-*co*-PEG3 copolymers within PBS with and without lipase over 35 days (*n* = 5).

The swelling ratio (%) of PGS and PGS-*co*-PEG copolymers in PBS at pH = 7.4 were calculated to evaluate bulk hydration characteristics (Fig. 5A and D). The maximum water uptake and equilibrium water content for all PGS-*co*-PEG3 copolymers occurred within 72 h, whereas for PGS alone this was reached after 24 h. However, the PGS-*co*-40PEG2 sample equilibrated after 168 h with 112.39 ± 3.70%, the equilibrium swelling ratio of PGS-*co*-60PEG3 was 119.77 ± 5.52% over 72 h. The addition of PEG2 and PEG3 led to significantly higher swelling (*p* < 0.001), and among PGS-*co*-PEG copolymers, the PGS-*co*-60PEG3 and PGS-*co*-40PEG2 achieved the highest swelling. Pure PGS showed a negligible water swelling ratio of 4.72 ± 0.59%, whereas PGS-*co*-40PEG2 and PGS-*co*-60PEG3 exhibited almost 23 and 25-fold increases in the water uptake, respectively. Therefore, the incorporation of PEG results in an increase in hydrophilicity, which directly relates to the concentration of PEG. Also, due to the larger swelling ratios, the PGS-*co*-PEG copolymers can be regarded as hydrogels.^15,42,43^ Comparing PGS-*co*-PEG2 and PGS-*co*-PEG3 hydrogels shows that similar incorporated amounts of PEG2 (20% and 40%) led to higher water uptake ratios. This phenomenon may be caused by the lower crosslinking density and lower molecular weight of PGS-*co*-PEG2 hydrogels.^15,44^

#### 3.6.2. pH-Responsive behaviours (swelling in pH = 5 and 9.1)

A diverse range of physiological functions and medical conditions within the human body result in a similar diversity of pH conditions. For example, the surface pH of healthy skin is 5.5, while the pH of a skin burn injury is between 9.5 and 10.5.^45^ Hence, hydrogels with pH-responsive properties have attractive potential in many biomedical and tissue engineering applications since these biomaterials can alter their structure and properties based on environments with different biological pH ranges.^46^ As discussed previously in the NMR and FTIR sections, 3.2 and 3.3, some hydroxyl and carboxyl groups were reacted to form covalent crosslinks in PGS-*co*-PEG; however, several ionic functional groups remained in these copolymer hydrogels. By deprotonation, the carboxyl (–COOH) and hydroxyl (–OH) groups can be ionised, becoming negatively charged carboxyl (–COO^−^) and hydroxyl (–O^−^) groups. As a result, the pH responsiveness of PGS-*co*-PEG hydrogels was evaluated by swelling assays at three different pHs, 5, 7.4 and 9.1, over 192 h. Fig. 5B and E show the water swelling ratio in the percentage of PGS-*co*-PEG hydrogels at acidic (citrate buffer, pH 5.0) and basic (NaOH–glycine buffer, pH 9.1) pH. Under acidic conditions, the PGS-*co*-PEG hydrogels, both PGS-*co*-PEG2 and PGS-*co*-PEG3, swelled less than under neutral conditions. The swelling ratios (%) at equilibrium measured at pH 5.0 were 4.36 ± 0.22% for PGS, 12.89 ± 2.17% for PGS-*co*-20PEG2, 65.22 ± 5.94% for PGS-*co*-40PEG2, 9.77 ± 0.54% for PGS-*co*-20PEG3, 32.43 ± 2.81% for PGS-*co*-40PEG3 and 65.78 ± 4.35% for PGS-*co*-60PEG3. However, the swelling ratio of samples at pH 9.1 enhanced significantly to 24.35 ± 3.98% for PGS-*co*-20PEG2, 180.16 ± 4.73% for PGS-*co*-40PEG2, 19.99 ± 1.47% for PGS-*co*-20PEG3, 92.24 ± 4.49% for PGS-*co*-40PEG3 and 186.80 ± 6.52% for PEG-*co*-60PEG3, whereas PGS swelling ratio (4.73 ± 0.40%) did change. Interestingly, the water swelling equilibria for the PGS-*co*-PEG copolymers were different at three measured pH conditions of 5, 7.4 and 9.1. It was determined that the swelling equilibrium at pH 7.4 was around 72 h, whereas, at pH 5.0 and 9.1, it took longer to reach equilibrium, taking 96–144 h. The swelling ratios of PGS-*co*-40PEG2 and PGS-*co*-60PEG3 at pH 9.1 were much higher than other conditions (Fig. 5B and E). In amphiphilic hydrogels like PGS-*co*-PEG, due to the resistive accumulation of hydrophobic polymer chains, the ionisation of functional groups can be stopped.^42,47,48^ When the hydrophilic segments of the amphiphilic structure are more dominant, the ionisation of carboxyl groups can occur much more easily. Therefore, PGS-*co*-40PEG2 and PGS-*co*-60PEG3, compared to other samples, were more hydrophilic owing to the greater amounts of PEG as it can provide much more free space and more chain relaxation to ionise carboxyl groups, causing a significantly higher swelling ratio of PGS-*co*-40PEG2 and PGS-*co*-60PEG3 at pH 9.1. A growing number of drug delivery systems have been developed using pH-responsive polymers, making PGS-*co*-PEG copolymers suitable for these applications.^49^

#### 3.6.3. Enzymatic degradation

*In vitro* degradation profiles of PGS-*co*-PEG2 and PGS-*co*-PEG3 samples in PBS with and without enzyme (lipase) were measured over the course of up to 35 days (Fig. 5C and F). The mechanism of enzymatic degradation of PGS-*co*-PEG is based on ester bond hydrolysis.^36^ It is evident from the degradation profiles that there is a direct relationship between the loaded amount of PEG and the enzymatic degradation rates. PGS-*co*-40PEG2 and PGS-*co*-60PEG3 exhibited the highest weight losses, which led to the remaining weight of 74.89 ± 1.16% and 63.32 ± 1.42%, respectively, over 35 days of incubation in the PBS (Fig. 5C and F). The presence of lipase resulted in the accelerated degradation kinetics of PGS-*co*-PEG2 and PGS-*co*-PEG3 specimens. Similarly, PGS-*co*-40PEG2 and PGS-*co*-60PEG3 showed the fastest enzymatic degradation among others, and the remaining weights were 53.97 ± 2.55% and 52.82 ± 1.35%, respectively (Fig. 5C and F). These two samples previously presented better hydration properties as measured by water swelling ratio that can be associated with greater degradation. From this data, it can be concluded that higher concentrations of PEG lead to greater ratios of swelling, and this can cause faster degradation rates since the higher loading of PEG results in greater hydrophilicity.^50^

It is worth mentioning that degradation profiles are significantly affected by pH.^51,52^ As previously mentioned, adding PEG, either PEG2 or PEG3, resulted in higher swelling at pH 9.1 > 7.4 > 5 and faster degradation in PBS at pH = 7.4 with and without enzyme. We observe a higher rate of hydrolysis in the PGS-*co*-PEG hydrogels at pH = 9.1 compared to pH = 5, which is correlated to a more rapid swelling behaviour in these hydrogels at high compared to low pH. The degradation profiles indicate an initial high degradation rate that slows down and correlates well with the high initial PEG release observed in Fig. 4B. After ∼15 days, both the PEG release and the degradation slow down, which indicates that the PGS hydrolysis is slower (likely because of increased hydrophobicity). The general higher degradation rate in basic compared to acidic environments can be explained *via* a respectively higher swelling rate in basic conditions. In acidic conditions (pH ∼ 5), the carboxylic acids that are remnant within the PGS-*co*-PEG structure will be protonated, while at higher pH, they are deprotonated. The charge build-up within the structure at high pH will result in a higher swelling and subsequent more rapid degradation of the PGS-*co*-PEG.^42,52^

In order to develop scaffolds that can withstand recurring dynamic loads and also provide suitable conditions for cell attachment, proliferation, and differentiation, it is essential to design degradable elastomers with mechanical and surface properties that mimic soft tissues.^53^ As a result, PGS-*co*-PEG copolymers are well suited to such applications.

### 3.7. Poly(ethylene glycol) (PEG2) and glycerol ethoxylate (PEG3) release study

PEG release from hydrogels was assessed to understand better the amount of crosslinking density using a colourimetric assay with a two-phase system that yields a visible purple-pink colouration at 510 nm if ferrothiocyanate-PEG complexes are introduced into the chloroform phase.^31,54^ All the samples that contained PEG released PEG into the solution as expected, and those specimens that included higher percentages of PEG resulted in greater amounts of PEG release (Fig. 4B). Also, all hydrogels containing PEG2 and PEG3, released less than 6% PEG in total, and this is indicative of high crosslinking density in samples after the thermal curing process. Over 35 days, the lowest PEG release was observed in PGS-*co*-20PEG3 with values of 0.94 ± 0.08% and 84.21 ± 0.08 μg mL^−1^, whereas the highest release was seen in PGS-*co*-60PEG3 with 5.88 ± 0.02% and 1492.13 ± 0.02 μg mL^−1^ (*p* < 0.0001). The PEG released from PGS-*co*-40PEG2 was close to PGS-*co*-60PEG3, and there was no significant difference between these samples; thus, it can be said they had similar profiles of PEG release. PGS-*co*-20PEG3 and PGS-*co*-20PEG2 also had similar profile releases, though there was a significant difference (*p* < 0.05) between the quantities and percentages released. Based on these results, it can be concluded that PGS-*co*-PEG2 had higher amounts and percentages of release, which can be related to the structure of PEG2 and the lower crosslinking density of these samples.

### 3.8. Uniaxial tensile test

**Fig. 6:**
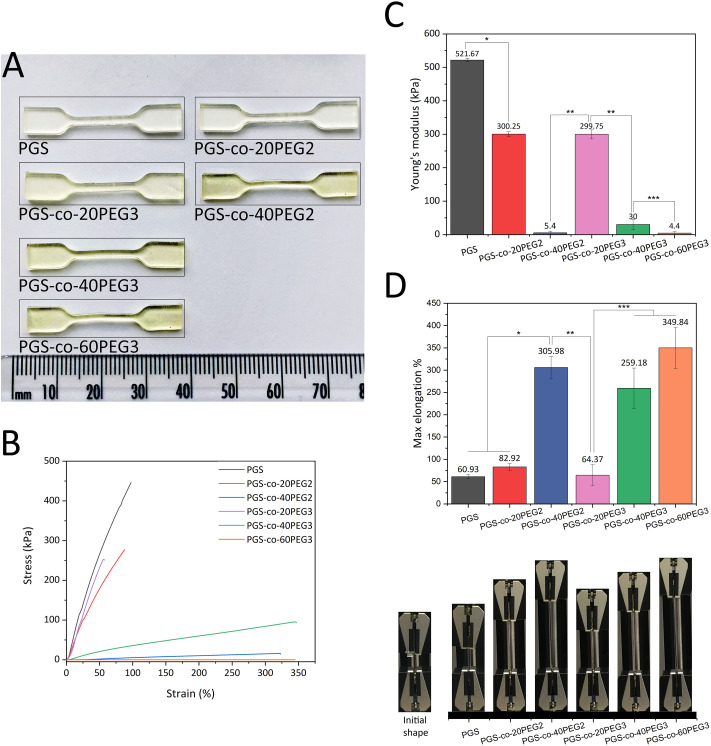
Mechanical properties of PGS-*co*-PEG2 and PGS-*co*-PEG3 (*n* = 5). (A) An image of fully cured PGS-*co*-PEG copolymer specimens with various PEG types and contents in dog-bone shape. Because of PEG addition, a colour change from pale to dark yellow was seen, (B) tensile stress–strain curves, (C) Young's modulus and (D) maximum elongation percentages and images of PGS-*co*-PEG2 and PGS-*co*-PEG3 copolymers, half of the tip of the clamp is 4 mm. Data are means ± SD (*, *** *P* < 0.001 and ** *P* < 0.01).

Previous studies have reported that the tensile strength of PGS and its derivatives have nonlinear stress–strain behaviour, and as a result, they are classed as soft elastomeric materials. These flexible elastomers can recover from large deformations due to the crosslinking and interactions between hydrogen bonds and hydroxyl groups within the PGS structure.^12,55^ Previous studies have shown that the addition of PEG2 into the backbone of PGS enhanced the elastomeric characteristics significantly, and the resulting PGS-*co*-PEG could tolerate excessive deformations with no friction or cracking.^36,40,56^ Synthesis parameters affect the degree of esterification, which in turn results in different physical, chemical and mechanical properties. Consequently, this study sought to investigate the effect of two types of PEG, PEG2 and PEG3, on PGS-*co*-PEG copolymers, for the first time. Comparing PGS-*co*-PEG2 and PGS-*co*-PEG3 samples, when the percentage of PEG is higher than 20%, PGS-*co*-PEG3 hydrogels containing PEG3 showed greater mechanical strength, PGS-*co*-40PEG3 > PGS-*co*-40PEG2 and PGS-*co*-60PEG3 ≃ PGS-*co*-40PEG2. Therefore, since PEG3, it is more reactive than PEG2, led to an increase in crosslinking density within the copolymer. Also, the mechanical properties results are consistent with the results of sol–gel content analysis (demonstrated in Table 3). In general, the addition of PEG decreased the mechanical strength of the copolymers, but increased tensile strains, and this resulted in greater flexibility. The resulting copolymers were extremely stretchable and bendable, particularly when higher PEG contents (40% and 60% wt) were used (Fig. 6 and ESI†). PGS-*co*-PEG2 and PGS-*co*-PEG3 featured highly elastomeric mechanical behaviours by reaching max elongation of 305.98 ± 24.60% and 349.84 ± 45.69% for PGS-*co*-40PEG2 and PGS-*co*-60PEG3, respectively, which were a more than five-fold increase compared to pure PGS (Fig. 6D). However, PGS-*co*-PEG2 hydrogels containing PEG2 had slightly greater elongation than PGS-*co*-PEG3. This feature allowed complex deformations such as stretching and knotting with PGS-*co*-PEG copolymers, including 40% and 60% PEG.

As expected, all PGS-*co*-PEG copolymers exhibited decreased tensile strength in comparison to pure PGS, though their ability to with stand tensile strains was increased (Fig. 6). Higher PEG concentrations, either PEG2 or PEG3, resulted in lower mechanical strength (Fig. 6). There are two key reasons for this: first, PEG introduces soft chain segments within the copolymer structure, which concomitantly reduces the percentage of rigid chain segments of PGS, and second, the presence of PEG in the structure causes a lower crosslinking density since fewer hydroxyls are available in the polymer backbone. Also, the addition of PEG reduces the degree of esterification and thus yields prepolymers with lower molecular weights.^36,38^ The given values of tensile strength of PGS-*co*-PEG copolymers are in the range of soft tissues like adipose or myocardial tissue, skin, nerve or cartilage which shows promise for soft tissue engineering.^4,42,57,58^

### 3.9. Lap-shear strength test

**Fig. 7:**
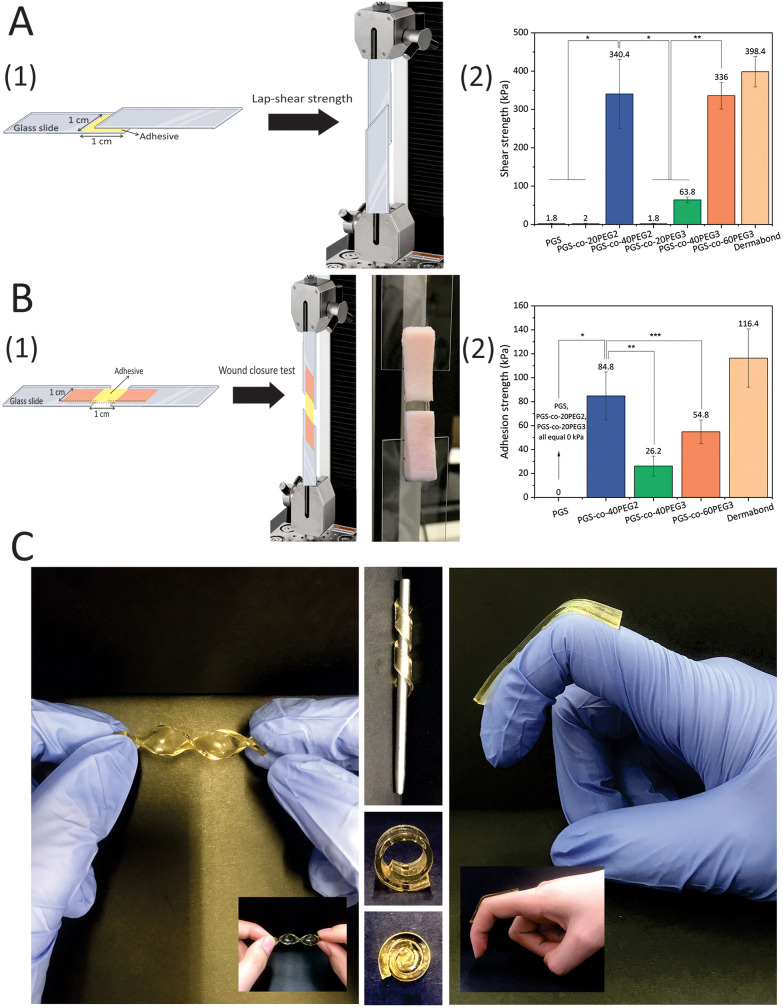
(A1) Schematic of the modified assay for lap-shear strength measurements (ASTMF2255-05) and (A2) average shear strengths of PGS-*co*-PEG adhesives (*n* ≥ 5) made of different PEG types and concentrations. Data are mean ± SD (*, ** *P* < 0.0001). (B) *In vitro* adhesion properties of PGS-*co*-PEG hydrogels using porcine skin as biological substrates. (B1) Schematic and real picture of the modified test for wound closure strength test (ASTM F2458-05) and (B2) average adhesive strengths of PGS-*co*-PEG adhesives (*n* ≥ 5) made of different PEG types and concentrations. Data are means ± SD (*, ** *P* < 0.0001, and *** *P* < 0.05). Dermabond™ mini topical skin adhesive was used as a control. (C) Images of PGS-*co*-40PEG2 and PGS-*co*-60PEG3 in different conditions and angles to show the flexibility of the copolymers.

A range of shear stresses was applied to samples, and these experiments showed appropriate controllability and repeatability of shearing characteristics (Fig. 7). The highest lap-shear strengths were for PGS-*co*-40PEG2 (340.4 ± 49.66 kPa) and PGS-*co*-60PEG3 (336.00 ± 35.07 kPa) (Fig. 7A1). These values were significantly higher than the lap-shear strength of other samples (*, ** *P* < 0.0001). Pure PGS and specimens containing 20% of PEG either PEG2 or PEG3 were not adhesive (Fig. 7B2); therefore, it can be concluded that PEG ≥ 40% resulted in a significant increase in lap-shear strength. Moreover, the increased lap-shear strengths of PGS-*co*-40PEG2 and PGS-*co*-60PEG3 were noticeably greater than the reported lap-shear strength of commercially available sealants or bioglues, such as Evicel (∼207 kPa) and CoSEAL (∼69 kPa).^33,59,60^ As a control, Dermabond™ mini topical skin adhesive was used, which had a lap-shear strength of ∼398 kPa. Adhesive polymers contain numerous hydrophilic groups, (*e.g.* hydroxyl, carboxyl, amide and sulphate), known as adhesively active groups, leading to attachments by hydrogen bonding and hydrophobic or electrostatic interactions. Also, these hydrophilic groups result in swelling in aqueous environments. PEG is FDA approved for many biomedical applications, for example it is widely used as a bioadhesive polymer in several applications like bioglue and drug delivery systems.^61,62^ Furthermore, one of the usual techniques to develop bioadhesives is to conjugate PEG chains to the specified molecules, *i.e.* PEGylation.^63^ Therefore, it can be concluded that the introduction of PEG to PGS resulted in adhesive properties and an increase in hydrophilicity.

### 3.10. Wound closure strength test

The adhesion strengths of the PGS-*co*-PEG adhesives were examined through a modified wound closure strength test, according to ASTM standard F2458-05 (Fig. 7). High adhesive strength was observed when PEG concentration was ≥40%, and the highest value was 84.80 ± 29.0 kPa for PGS-*co*-40PEG2 (Fig. 7). Notably, PGS-*co*-40PEG3 (∼26 kPa) and PGS-*co*-60PEG3 (∼54 kPa) reached higher adhesive strengths than those reported for CoSEAL (∼19 kPa) and Evicel (∼26 kPa).^33^ Furthermore, the measured strengths are within or greater than those of commercial bioadhesives or sealants like Quixil (24.6 kPa), Beriplast (24.2 kPa), Tachosil (59.6 kPa), and Tisseel (77.5 kPa).^64^ The control was Dermabond™ mini topical skin adhesive, which reached ∼116 kPa adhesion strength. High adhesion is an essential property for a bioglue or skin plaster because it prevents detaching from the target site and promotes biointegration.^59,65^ In addition, bioadhesives can be applicable in different fields such as wound healing, drug delivery, biosensor and tissue adhesive.^66,67^

### 3.11. Adhesion test with 100 g weight and flexibility

**Fig. 8:**
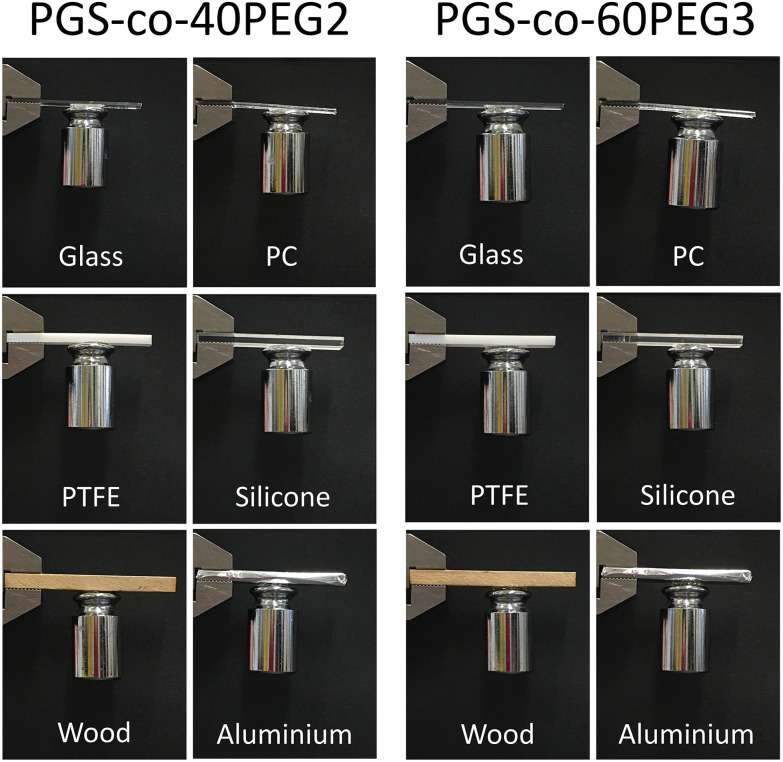
Pictures of various surfaces adhered to a 100 g stainless-steel weight by PGS-*co*-40PEG2 and PGS-*co*-60PEG3. These two copolymers illustrated strong adhesion to stainless steel, glass, polycaprolactone (PC), polytetrafluoroethylene (PTFE), silicone, wood and aluminium. The diameter of the samples was 12 mm.

PGS-*co*-40PEG2 and PGS-*co*-60PEG3 demonstrated the highest bioadhesive capacity; therefore, these samples were chosen to examine their adhesion properties with various substrates. These two PGS-*co*-PEG samples showed quick and strong adhesion to substrates including glass, polycaprolactone (PC), polytetrafluoroethylene (PTFE), silicone, wood and aluminium. As can be seen in Fig. 8, both PGS-*co*-40PEG2 and PGS-*co*-60PEG3 can support a weight of 100 g by adhering a stainless steel to various substrates. The time required for adhesion to each substrate varied, although in general, it was around 5 minutes. In addition, these two copolymers, PGS-*co*-40PEG2 and PGS-*co*-60PEG3, were more flexible compared to other samples, which, since they are transparent, light weight and flexible, might have suitable medical applications such as wound dressing^68^ or non-medical application for as adhesive sensors or for flexible devices (Fig. 7C and ESI†).^69^ Furthermore, it is possible for flexible materials to sense and adapt to changes in the microenvironment; such materials are capable of improving cellular mechanosensing by dynamically adjusting their topography and local mechanics.^70^

### 3.12. Cell metabolic activity

**Fig. 9:**
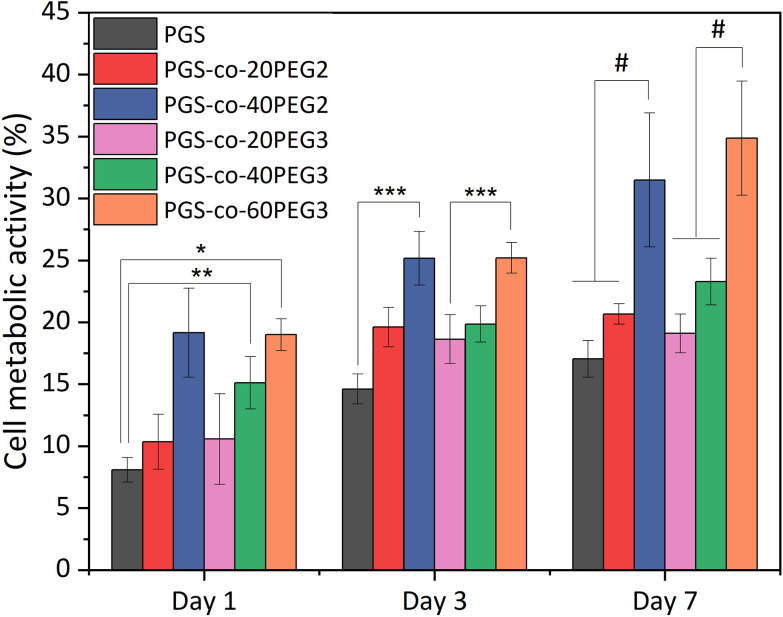
Results of *in vitro* cell metabolic activity. The results are reported in percentage and normalised by positive control, tissue culture plate, from the resazurin assays of PGS-*co*-PEG for either PEG2 or PEG3 at day 1, 3 and 7 (*n* = 5; *, **, *** *p* < 0.01 and # *p* < 0.001).

The *in vitro* cell metabolic assays were performed on PGS-*co*-PEG2 and PGS-*co*-PEG3 with resazurin dye and human keratinocyte (HaCaT) cell lines for up to 7 days. There was no evidence of a decrease in metabolic activity for cells cultured on PGS-*co*-PEG2 and PGS-*co*-PEG3 (Fig. 9), suggesting that the samples were not cytotoxic. Between day 1 and day 7, an increase in cell metabolic activity was observed on PGS-*co*-PEG2 and PGS-*co*-PEG3, indicative of cell proliferation, with the maximum observed on day 7, likely coinciding with saturation of cells on the sample. The highest proportional changes in cell metabolic activity were observed in assays with PGS-*co*-40PEG2 and PGS-*co*-60PEG3, with increases from 19.15 ± 3.59% to 31.49 ± 5.41% in PGS-*co*-40PEG2, and from 18.99 ± 1.28% to 34.86 ± 4.6% in PGS-*co*-60PEG3 between day 1 and day 7. Cell metabolic activity was significantly enhanced at each interval in correlation with higher PEG concentration and higher hydrophilicity (*, **, *** *p* < 0.01 and # *p* < 0.001) (Fig. 9). In addition, a two-way analysis of variance (ANOVA), revealed significant differences between day 1 and day 3 (*p* < 0.05). The measured metabolic activity of cells grown on both PGS-*co*-PEG2 and PGS-*co*-PEG3 copolymers, including PEG ≥ 40%, was greater than those of PGS alone. PGS has been investigated for various tissue engineering applications due to its biocompatibility both *in vitro* and *in vivo*;^11,71,72^ hence, the results of this work demonstrate that PGS-*co*-PEG, including PEG ≥ 40%, are also suitable candidates for use in biomedicine as they have good biocompatibility relative to PGS.

### 3.13. Live/dead assay

**Fig. 10:**
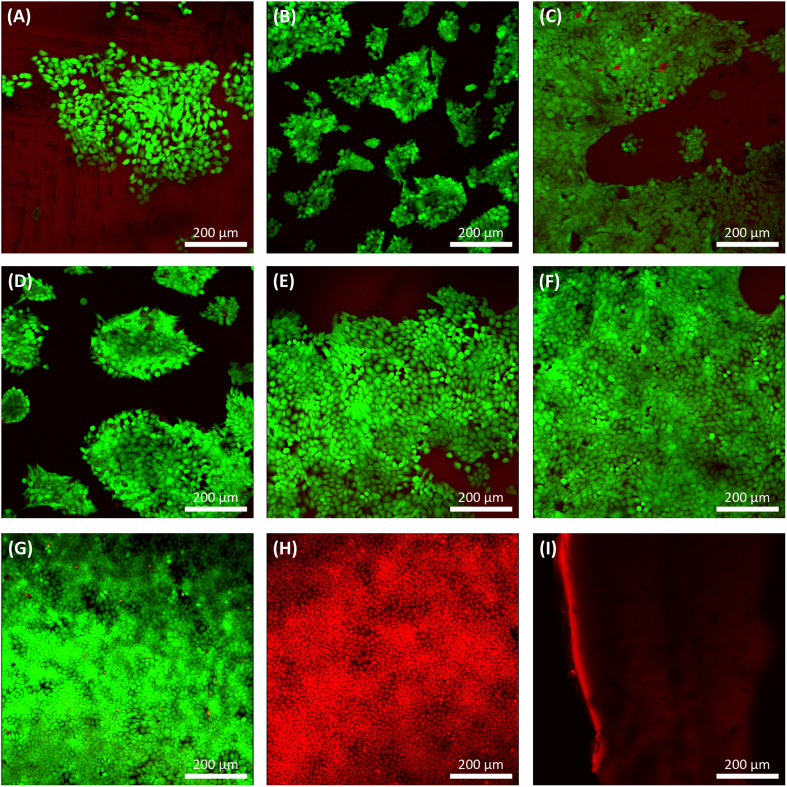
Confocal images of live/dead assay stained living cells green and dead cells red. Live/dead results of seeded HaCat cells on (A) PGS, (B) PGS-*co*-20PEG2, (C) PGS-*co*-40PEG2, (D) PGS-*co*-20PEG3, (E) PGS-*co*-40PEG3 and (F) PGS-*co*-60PEG3 on day 3. Live/dead results of (G) seeded HaCat cells on tissue culture plastic on day 3, (H) dead HaCat cells on tissue culture plastic on day 3 (killed by ethanol 100% for 15 min) and (I) stained PGS/PGS-*co*-PEG sample with live/dead with no cells. Scale bar: 200 μm.

After three days of culture, a live/dead assay was used to determine cell viability, morphology, and adhesion of grown HaCat cells on samples (Fig. 10). Seeded HaCat cells on tissue culture plastic within two conditions, culturing HaCat cells for three days (Fig. 10G) and killing HaCat cells after three days of culture by ethanol 100% for 15 min (Fig. 10H), used as controls. Also, PGS and PGS-*co*-PEG with no cells were stained by live/dead again as a control (Fig. 10I) since the pale red background can be seen under confocal imaging due to the low fluorescence of the non-specific absorbance of ethidium bromide onto the PGS and/or PGS-*co*-PEG.

PGS, PGS-*co*-PEG2 and PGS-*co*-PEG3 all showed live HaCat cells (∼95%) in a more cuboidal shape with few dead on day 3 (Fig. 10). Those copolymers with higher PEG concentrations represented enhanced cell affinity as a result of increased PEG incorporation, leading to favourable cell adhesion. Surface wettability was increased when PEG was copolymerised, which is a precursor to cell attachment, and can explain the enhanced adhesion of cells to PGS-*co*-PEG elastomers. Similarly, the resazurin results represented the same trend: a higher concentration of PEG led to greater HaCat cell metabolic activity. Thus, cell metabolic activity and live/dead assay results showed that PGS-*co*-PEG copolymers could support cell attachment and proliferation, making them suitable for various biomedical applications.

## 4. Conclusion

In summary, it is possible to modify synthetic polymers in to be used for broader biomedical applications. Among synthetic polymers applicable to biomedicine, PGS is a potential novel candidate that increasingly studied as a potential biomaterial for soft tissue engineering. A condensation copolymerisation method was successfully used to prepare PGS-*co*-PEG copolymer hydrogels with different types of PEG, either PEG2 or PEG3, and weight ratios. The PGS-*co*-PEG elastomers were characterised and optimised based on their physiochemical and biocompatibility properties. It was determined that PGS-*co*-PEG had improved hydrophilicity by decreasing the water contact angle and increasing the water swelling ratio, which resulted in swollen copolymer hydrogels. As a result, the cell metabolic activity was significantly enhanced by increasing hydrophilicity compared to PGS alone. The PGS-*co*-PEG copolymers displayed excellent elastomeric properties that can withstand stretching and bending mechanical deformations. Due to the ionisable functional groups, PGS-*co*-PEG hydrogels displayed pH-responsive behaviour in terms of water absorption at acidic, neutral and basic pH values. The PGS-*co*-PEG elastomers illustrated accelerated enzymatic degradation compared to PGS, and their degradation was controllable and adjustable *in vitro* with PBS and PBS with lipase. Also, these copolymers showed remarkable bioadhesiveness compared to commercial bioglues, which was measured by shear strength and wound closure strength assays. This study demonstrates a promising approach for exploiting multifunctional PGS-*co*-PEG copolymers for applications in soft biomaterials, tissue engineering, and drug delivery systems.

## Author contributions

Mina Aleemardani: conceptualization, investigation, methodology, validation, funding acquisition, writing – original draft. Michael Zivojin Trikić: investigation, methodology, supervision, writing – review & editing. Nicola Helen Green: conceptualization, investigation, methodology, validation, funding acquisition, supervision, writing – review & editing. Frederik Claeyssens: conceptualization, investigation, methodology, validation, funding acquisition, supervision, writing – review & editing.

## Conflicts of interest

The authors declare no conflict of interest.

## Supplementary Material

BM-010-D2BM01335E-s001

BM-010-D2BM01335E-s002

BM-010-D2BM01335E-s003
